# Production and characterization of *Aspergillus niger* GH29 family α-fucosidase and production of a novel non-reducing 1-fucosyllactose

**DOI:** 10.1007/s10719-019-09896-w

**Published:** 2019-12-02

**Authors:** Anne Usvalampi, Marcela Ruvalcaba Medrano, Hannu Maaheimo, Heidi Salminen, Olli Tossavainen, Alexander D. Frey

**Affiliations:** 1 Department of Bioproducts and Biosystems, Aalto University School of Chemical Engineering, P.O. Box 16100, Espoo, Finland; 2 VTT Technical Research Centre of Finland Ltd., P.O. Box 1000, Espoo, Finland; 3 Valio Ltd., P.O.Box 10, Helsinki, Finland

**Keywords:** Fucosyllactose, α-Fucosidase, *Aspergillus niger*, Transfucosylation, 1-Fucosyllactose, Non-reducing sugar

## Abstract

Fucosylated oligosaccharides are interesting molecules due to their bioactive properties. In particular, their application as active ingredient in milk powders is attractive for dairy industries. The objective of this study was to characterize the glycosyl hydrolase family 29 α-fucosidase produced by *Aspergillus niger* and test its ability to transfucosylate lactose with a view towards potential industrial applications such as the valorization of the lactose side stream produced by dairy industry. In order to reduce costs and toxicity the use of free fucose instead of environmentally questionable fucose derivatives was studied. In contrast to earlier studies, a recombinantly produced *A. niger* α-fucosidase was utilized. Using pNP-fucose as substrate, the optimal pH for hydrolytic activity was determined to be 3.8. The optimal temperature for a 30-min reaction was 60 °C, and considering temperature stability, the optimal temperature for a 24-h reaction was defined as 45 °C For the same hydrolysis reaction, the kinetic values were calculated to be 0.385 mM for the K_M_ and 2.8 mmol/(mg*h) for the V_max_. Transfucosylation of lactose occurred at high substrate concentrations when reaction time was elongated to several days. The structure of the product trisaccharide was defined as 1-fucosyllactose, where fucose is α-linked to the anomeric carbon of the β-glucose moiety of lactose. Furthermore, the enzyme was able to hydrolyze its own transfucosylation product and 2′-fucosyllactose but only poorly 3-fucosyllactose. As a conclusion, α-fucosidase from *A. niger* can transfucosylate lactose using free fucose as substrate producing a novel non-reducing 1-fucosyllactose.

**Electronic supplementary material:**

The online version of this article (10.1007/s10719-019-09896-w) contains supplementary material, which is available to authorized users.

## Introduction

α-L-fucose (Fuc) is an important building block of many biologically active oligosaccharides. It is frequently found at the non-reducing termini of various glycans, such as blood group antigens, human milk oligosaccharides and cell surface receptor proteins in the intestinal mucus layer. These terminal fucosyl residues play important roles in mammalian cell-to-cell communication and host-microbe interactions [1].

Fucosylation of intestinal epithelial cells seems to be a result of a symbiosis between bacteria and the host. The fucosylation has been shown to be a response to the colonization of the gut by certain commensal bacteria, such as *Bacteroides thetaiotamicron* and *Bacteroides fragilis.* These bacteria can utilize the fucosylated glycans as energy source, and can use this as a way to ensure a good carbon source for their own growth. In order to exploit the carbon source, these bacteria secrete fucosidases that cleave fucose from mucosal glycans, and while they utilize the rest of the glycan as energy source, free fucose becomes available for use also by other bacteria. Utilization of fucose as energy source by bacteria leads to the production of metabolites useful for the host, such as short chain fatty acids [2, 3].

Fucose-containing oligosaccharides could have several applications for example in prebiotics. Many bacteria and viruses use glycoconjugates, especially fucose, for lectin-mediated adhesion [4]. Exogenously supplied fucose can be used as a decoy for these pathogens. Instead of intestinal cells, the pathogens bind to soluble glycans and are washed away [5]. Free fucose in the intestines may also function as a signal molecule to warn about breakage of the mucosal barrier by mucin-hydrolyzing bacteria. This signaling mechanism could potentially be used as a means to educate the host’s mucosal immune system [6].

α-Fucosidases can catalyze the release of terminal fucose residues that are α-linked to glycans. α-Fucosidases are classified into three glycosyl hydrolase families, GH29, GH95 and GH151 (Carbohydrate Active Enzymes Database, www.cazy.org [7]). Currently, the GH151 family contains only three characterized members, and they seem to have poor activity on fucosylated substrates questioning whether they are genuine α-fucosidases [7–9]. Enzymes of the GH95 family, are highly specific for the hydrolysis of non-reducing terminal L-fucose linked to D-galactose residue by an α-1,2-linkage and are characterized by an inverting mechanism of hydrolysis [10]. Enzymes from the family GH29 catalyze the hydrolysis of α-L-fucosyl residues by retaining the configuration of the substrate anomeric center in the resulting product and can hydrolyze different types of glycosidic linkages. They commonly hydrolyze α-1,2-linkages between fucose and galactose or α-1,3 and α1,6-linkages between fucose and N-acetylglucosamine residues [7]. Family GH29 α-fucosidases have been subclassified into two groups. The subfamily A contains α-fucosidases with relatively relaxed substrate specificities, able to hydrolyze pNP-fucose, while the members of subfamily B are specific to α-1,3/4-glycosidic linkages and are practically unable to hydrolyze pNP-fucose [11].

Moreover, some α-fucosidases are known to have also transglycosylation activity, adding fucose units to a saccharide structure. All of the transglycosylating α-fucosidases belong to the GH29 family [12–14]. Transglycosylation activities are of interest as they can be used to synthesize oligosaccharides without the need for nucleotide-activated sugars as donor molecules.

The genome of *Aspergillus niger* CBS 513.88 encodes three different α-fucosidases, one belonging to family GH29 and two to family GH95, respectively [15, 16]. The *A. niger* GH29 α-fucosidase was chosen for this study as it has been reported to have one of the highest transglycosylating activities of all native α-fucosidases [13, 17]. However, so far, only partially purified enzyme preparations have been used. In this study, we characterized the recombinantly produced GH29 α-fucosidase and studied the transglycosylation reaction using natural sugars as substrate. We show that a new non-reducing 1-fucosyllactose is produced.

## Materials and methods

### Cloning of α-fucosidase

The gene encoding the C-terminally his-tagged α-fucosidase from *A. niger* CBS 513.88 (790 aa, accession number XP_001396349.2) was codon-optimized for expression in *Pichia pastoris* and obtained from GenScript cloned into pPICZ-alpha replacing the native signal sequence (AA 1 to 16) with the Mating factor alpha signal sequence. The resulting plasmid was named pPFucA-L. Based on an earlier gene annotation encoding a 531 aa long protein (accession number XP_001396349.1), a shorter version was generated using PCR. Oligonucleotides OMR1 (5′- GCA*GAATTC*ATGCCAGGTATTAGA-3′) and OMR2 (5’-GCG*TCTAG*ATTAATGATGGTGGTG-3′) were used to amplify the coding sequence of the shorter version using pPFucA-L as template. The PCR product was inserted into plasmid pPFucA-L using *EcoRI* and *XbaI* replacing the coding region of the long version. The resulting plasmid was named pPFucA-S harboring the C-terminally His-tagged short version. Plasmid pPFucA-S was verified by sequencing.

The plasmids were linearized using *SacI* and transformed into *P. pastoris* strains SMD1168H (Mut^+^ strain) and KM71H (Mut^S^ strain) (ThermoFisher) by electroporation. Colonies were selected on zeocin-containing plates and presence of the gene was confirmed using colony PCR.

### α-Fucosidase production

Colonies of *P. pastoris* transformants were grown and induced according to instructions in *Pichia* Expression Kit Manual (Invitrogen). In order to choose the best producing clones, α-fucosidase was produced in 2-L flasks using 200 mL of BMMY supplemented with 0.5% methanol every 24 h at 28 °C until activity increase came to a stop. Alternatively, the production was done in 2-L or 5-L bioreactors using *Pichia* fermentation process guidelines by Invitrogen, with minor modifications. In short, Fermentation Basal Salts medium supplemented with PTM_1_ was inoculated with 10% shake flask culture grown to OD 2–6. The cells were grown at 25 °C, pH 5 until all glycerol was consumed. Cell biomass was increased with glycerol fed-batch phase feeding 18 ml/h/L of 50% glycerol. After this, methanol fed-batch phase was started with 3.6 ml/h/L methanol feed. After 5 h the methanol feed was raised to 6.5 ml/h/L for maximal protein production.

The culture supernatant was cleared by centrifugation and the cleared supernatant from the production experiments was concentrated and buffer was changed by ultrafiltration using Pellicon BioMax 5 kDa filter units (Merck) and Vivaspin 20 ultrafiltration centrifuge tubes (MWCO 30,000 Da, Sartorius). The supernatant was concentrated 250 times and stored at +4 °C. Purity of the protein was checked using SDS-PAGE.

### Enzyme activity measurement

α-Fucosidase activity was measured using 4-Nitrophenyl α-L-fucopyranoside (pNP-fucose) as substrate. Reactions were performed in 100 μl using 50 mM Na-acetate buffer pH 5.0 and 2.5 mM pNP-fucose. After a 30-min incubation at 37 °C, 200 μl of 1 M glycine buffer pH 9.0 was added and absorbance was measured at 410 nm. All the assays were performed in quadruplicate.

The optimal pH was determined using glycine buffer between pH 2.2 and 3.3, Na-acetate buffer between 3.6 and 5.0 and Na-phosphate buffer between 6.0 and 7.0. The activity was measured after a 30-min reaction at 37 °C at given pH. The optimum temperature was determined for a 30-min reaction at temperatures between 30 and 70 °C with 5 °C increments at pH 5. Temperature stability of the enzyme was determined at 30 to 65 °C with 5 °C increments. The enzyme was incubated for 24 h at the indicated temperatures and the residual activity was measured for a 30-min reaction at pH 5 and 37 °C. For the enzyme kinetic studies, Na-acetate buffer at pH 3.6 was used at 45 °C and concentration of pNP-fucose was varied between 0.05 and 3 mM. Samples were taken and measured at 5-min intervals for 30 min.

### Western blotting

Cell extracts were prepared by vortexing with glass beads in PBS-buffer supplemented with 1 mM PMSF, 1 mM EDTA and 5% glycerol. The SDS-PAGE and Western blotting were performed according to general procedures. Anti-His antibody (Qiagen) was used in 1:1000 dilution. Rabbit-anti-mouse IgG coupled with HRP (Sigma-Aldrich) was used as the secondary antibody in 1:5000 dilution. Chemiluminescent detection reaction was performed using SuperSignal West Pico Chemiluminescent Substrate (ThermoFisher Scientific).

### Determination of N-glycosylation

N-glycosylation status of the purified protein was determined by mixing 2 μg of α-fucosidase with 2 μl 10X GlycoBuffer 3 (New England Biolabs) and 1 μl of Endoglycosidase H (Endo H) in a final reaction volume of 20 μl. The same amount of α-fucosidase enzyme was mixed with 1 μl of jack bean α-mannosidase enzyme in a final reaction volume of 20 μl in 50 mM sodium citrate buffer pH 5. Samples were incubated overnight at 37 °C. Control reactions without addition of enzymes were carried out. Activity of deglycosylated α-fucosidases was measured from 1:500 dilution and molecular weights were estimated using SDS-PAGE.

### Transglycosylation reaction

Transglycosylation reaction between fucose and lactose was performed at 50 °C using 100 g/L fucose and 200 g/L lactose and 140 U/L α-fucosidase in 50 mM ammonium acetate buffer pH 4.0. The reaction was run for 7–11 days after which it was stopped by incubating at 100 °C for 5 min. The reaction mixture was pasteurized at 80 °C for 15 min prior to the addition of the enzyme.

The product of the transglycosylation reaction was purified using preparative scale gel chromatography (Bio-Gel P-2, 2.5 × 100 cm, Bio-Rad). A solution containing approximately 500 mg of sugars was purified per batch. The solution was injected to the column and eluted with deionized water at 0.25 ml/min flow rate at room temperature using Äkta instrument (GE Healthcare). After a void volume of 200 ml, 40 fractions of 5 ml each were collected.

### Hydrolysis

The ability of α-fucosidase to hydrolyze different fucosyllactoses (FL) was tested with 2’-FL and 3-FL (Carbosynth) and the purified product from transglycosylation reaction. Approximately 500 ppm sugar was used in 50 mM ammonium acetate buffer pH 4.0 and enzyme was added to a final concentration of 44 U/L. The reaction was carried out at 50 °C using a rolling platform. Samples were taken for 24 h and the reaction was stopped by incubating at 100 °C for 5 min.

### MALDI-TOF MS

Tryptic peptides of Endo H treated concentrated samples were produced using In-Gel Tryptic Digestion Kit (Thermo Scientific) according to manufacturer’s instruction. Peptides were purified using C18 ziptips and eluted in 80% ACN, 0.1% TFA. Equal amounts of the sample and matrix (20 mg/ml of super-DHB in 30% ACN, 0.1% TFA or 20 mg/ml of HCCA in 30% ACN, 0.1% TFA) solution were mixed and 0.5 μl of the mixture was spotted on a target plate. Spectra were recorded using MALDI-TOF/TOF (UltrafleXtreme, Bruker Daltonics) operated in the positive ion reflector mode.

### Trisaccharide characterization

Prior to any analytical procedures, the samples were treated with Envi-18 solid phase extraction columns (Supelco, Sigma-Aldrich). The column (100 mg bed weight) was washed twice with acetonitrile and then equilibrated twice with water. The sample was loaded to the column in water and the column was washed with one sample volume of water. The flow-throughs were combined and filtered through a 0.2 μm filter.

#### LC-MS

LC-MS measurements were done as described in [18] with 10 μl injection volumes. In short, an HPLC (1200 Infinity, Agilent, Santa Clara, CA, USA) equipped with XBridge Amide Column (3.5 μm particle size, 2.1 × 150 mm, Waters, Milford, MA, USA) was used. Buffers were A) 70/30 Milli-Q H_2_O/Acetonitrile supplemented with 0.1% NH_4_OH and B) 80/20 Acetonitrile/Milli-Q H_2_O supplemented with 0.1% NH_4_OH. The gradient used was 0% to 60% buffer A in 15 min, then 60% to 0% buffer A in 3 min. The sugars were detected using Q-TOF MS (6530, Agilent, Santa Clara, CA, USA) in the negative ion mode. Lactose, 2’-FL and 3-FL were used as standards in concentrations between 1 ppm to 100 ppm. This LC setup was also used to purify the sugar for NMR studies, with the following modifications: The gradient was 0% to 60% buffer A in 10 min, then 60% to 0% buffer A in 1 min and equilibration of 4 min between injections. Injections of 35 μl containing approximately 3000 ppm of the product were used and the fucosyllactose fraction (time 8 min to 10 min) was collected prior to the MS-detector.

#### HPAEC-PAD

An ICS-5000 system (Dionex) combined with CarboPac PA1 column (4 × 250 mm, Dionex) was used to analyze sugar concentrations in hydrolysis reactions. Column temperature was kept at 30 °C. 100 mM NaOH (A) and 1 M NaOAc in 100 mM NaOH (B) were used as eluents. The separations were performed at a flow rate of 1 ml/min. The starting conditions were 100% A followed by a 25 min gradient to 40% A and 60% B. The column was washed with 100% B for 10 min and equilibrated with 100% A for 10 min. 10 μl injection volume was used. Chromeleon 7.2 software (Thermo Scientific) was used for data processing. 2’-FL and 3-FL were used as standards.

#### NMR spectroscopy

The LC purified carbohydrate sample was dissolved in 600 μl of D_2_O containing 0.05% 3-(trimethylsilyl)propionic-2,2,3,3,d4 acid (Aldrich, St. Louis, MO, USA). All NMR experiments were carried out at 22 °C on a 600 MHz Bruker Avance III NMR spectrometer equipped with a QCI H-P/C/N-D cryoprobe and 1D and 2D NMR spectra were recorded as described earlier [18]. In addition, pure shift HSQC spectra [19] were recorded using Bruker’s pulse program hsqcetgpsp.2_bbhd in which the ^1^H broadband homodecoupling during acquisition is achieved with a [BIRD]r,x element.

## Results

### α-Fucosidase production

Two different annotations for the gene encoding the GH29 α-fucosidase from *A. niger* are reported in publically available databases and so far no direct experimental evidence exists corroborating any of the two annotations. The longer version encodes a protein of 790 amino acids while the shorter one of 531 residues. The 259 additional amino acids are exclusively situated in the N-terminus of the enzyme, while the rest of the enzyme is identical. Both protein versions share the conserved domain of α-fucosidase from amino acid 346 to 696 according to sequence of the long version. Up to date, there is no information about the putative function of the additional 259 amino acids in the N-terminus except for the 16 amino acids long signal sequence. No amino acid sequence functioning as secretion signal peptide can be predicted in the short annotation implicating that it would encode an intracellular enzyme. Thus, we wanted to experimentally establish, which of the two gene annotations encodes the functional enzyme.

The two constructs encoding short or long version of the enzymes were transformed into Mut^S^ and Mut^+^ strains of *P. pastoris* for the initial enzyme production. Colonies from each transformation were grown in order to produce α-fucosidase. No activity of the short version of the α-fucosidase was detected in neither supernatant or cell extracts (data not shown). In addition, no band of appropriate size was detected in cell extracts or supernatants using SDS-PAGE or Western blotting, indicating that the short version of the enzyme was either not produced or was degraded right after the expression (data not shown). Strains expressing the short version were therefore excluded from further studies. We were able to produce active α-fucosidase enzyme in strains of both genetic backgrounds transformed with the pPFucA-L plasmid. Due to previous experience in our laboratory, shake flask cultivations were done using the Mut^S^ strain, while bioreactor cultivations were performed using Mut^+^ strain. In shake flasks cultivations the highest α-fucosidase activity was observed after 7 days of cultivation and reached 96 U/L. Bioreactor cultivations were done in 2-L and 5-L reactors. The glycerol growth phase lasted for 28 h after which the expression phase with a constant methanol feed was continued for another 80 h. The α-fucosidase activity in the supernatant reached 250 U/L.

**Fig. 1:**
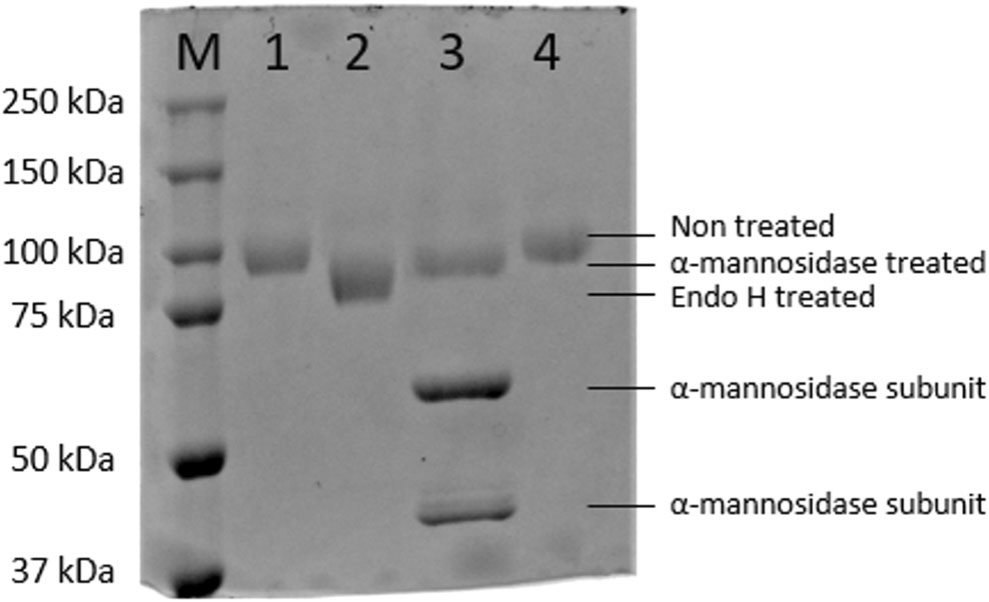
SDS-PAGE of α-fucosidase produced in *P*. *pastoris*. The enzyme was treated with Endo H or α-mannosidase overnight at 37 °C. In control samples, water was added instead of the enzymes. Samples were loaded onto 7.5% SDS-PAGE and gels were stained with Commassie Blue. Lane M, protein standard; lane 1, Endo H control; lane 2, Endo H treated α-fucosidase; lane 3, α-mannosidase treated α-fucosidase; lane 4, α-mannosidase control. The location of the different enzyme forms is indicated on the right side of the gel. Two major bands corresponding to α-mannosidase subunits (44 kDa and 66 kDa) appear in lane 3

After the production phase, the culture supernatant was cleared by centrifugation and concentrated by ultrafiltration. SDS-PAGE analysis was done to check the purity of the enzyme. The concentrated enzyme was >95% pure as no other bands were detected on Coomassie stained SDS-PAGE gels (Fig. 1 lanes 1 and 4). Interestingly, the apparent molecular weight of the α-fucosidase preparations ranged between 96 kDa and 150 kDa for the enzyme produced in shake flasks and in bioreactors, respectively. It seems that the enzyme produced in bioreactors with Mut^S^ strain are glycosylated to a higher degree than the enzyme produced in shake flask cultivations with Mut^+^ strain.

In order to confirm the identity of the recombinantly produced α-fucosidase, the enzyme was digested with trypsin and a peptide fingerprint of the tryptic peptides was generated. Overall, 51 peptides corresponding to the α-fucosidase protein sequence were sequenced and identified yielding a 61% coverage of the complete protein sequence (Fig. S1 and Table S1 in Online Resource). Furthermore, the obtained peptide fingerprint also indicated that the full-length protein according to the long annotation is produced and the N-terminus is correctly processed. Overall, we concluded that the concentrated protein is the recombinant α-fucosidase and it is sufficiently pure. Furthermore, the concentrated culture supernatant does not contain any other contaminating proteins.

### Degree of N-glycosylation affects the enzymatic activity

As the extent of N-glycosylation, respectively the degree of hypermannosylation, varies between different fungal expression hosts, we explored how the activity of the recombinant α-fucosidase was affected. In order to deglycosylate the enzyme, it was incubated with either Endo H or α-mannosidase enzymes under non-denaturing conditions. Endo H cleaves accessible N-glycans within the chitobiose core removing the complete N-glycan except the N-linked GlcNAc residue. In contrast, jack bean α-mannosidase removes α-linked mannoses from the non-reducing end, reducing the degree of hypermannosylation.

Each treatment was done in triplicate and after the digestion, the samples were visualized using SDS-PAGE (Fig. 1). The apparent size of the enzyme was reduced from an estimated molecular weight of 96 kDa (lane 1 Endo H control and lane 4 α-mannosidase control) to about 85 kDa using Endo H (lane 2) and 92 kDa using α-mannosidase (lane 3), respectively. As the theoretical molecular weight of this α-fucosidase is 87.1 kDa, the results indicated that the N-glycans are completely accessible and can be removed using Endo H. In order to compare how the extent of glycosylation affects activity, an activity assay was performed using the deglycosylated enzymes. The relative activities of α-mannosidase and Endo H treated samples were 106% and 112% of the activity compared to the untreated sample. Based on these observations we concluded that the higher degree of N-glycosylation decreased the hydrolytic activity of the α-fucosidase.

### Profiling of the hydrolytic activity

**Fig. 2:**
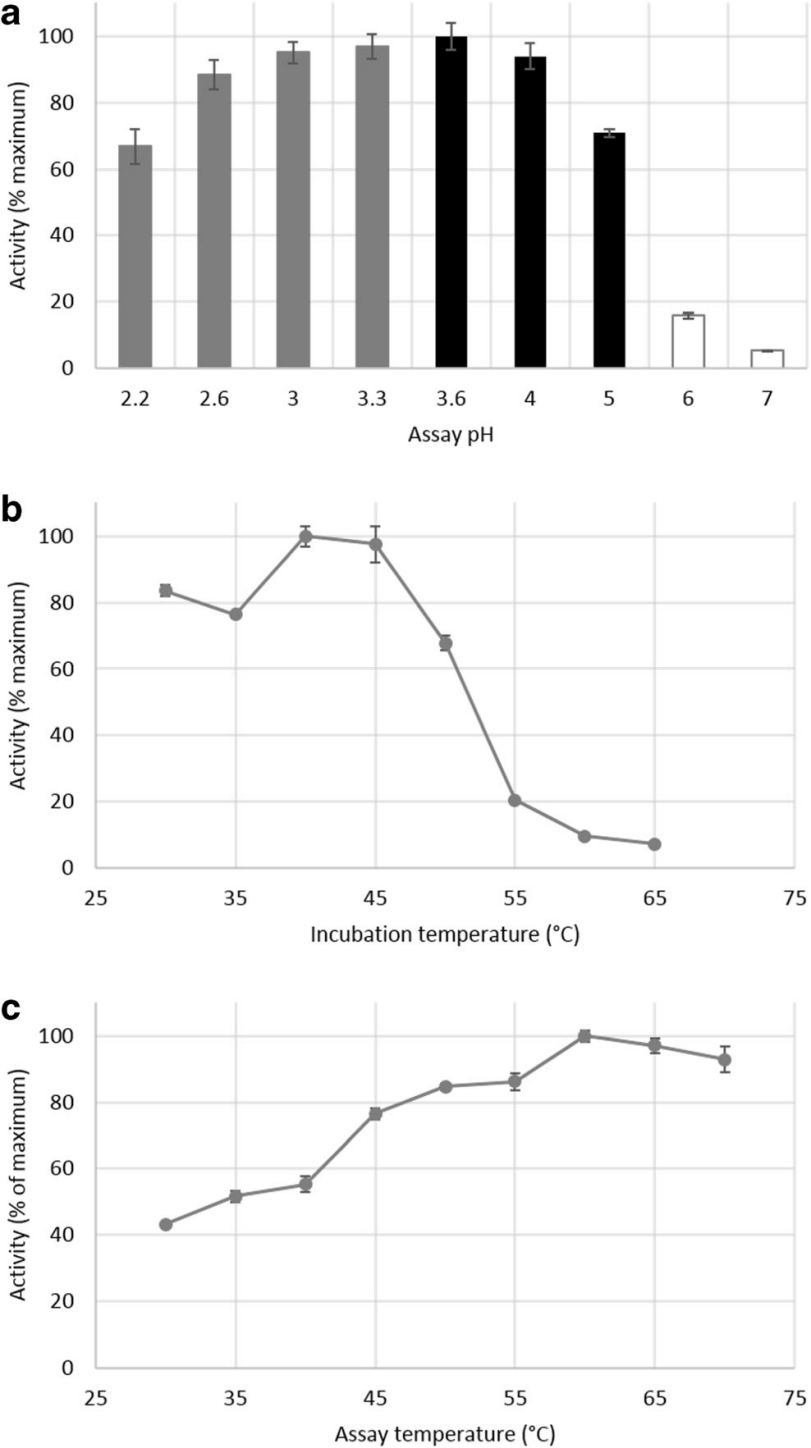
**a** Effect of pH on hydrolytic activity of α-fucosidase. Grey: Glycine buffer, Black: Acetate buffer, White: Phosphate buffer. **b** Thermal stability of α-fucosidase at different temperatures for a 24-h reaction. **c** Effect of temperature on the hydrolytic activity of α-fucosidase for a 30-min reaction. pNP-fucose was used as substrate in all tests. All the assays were done in quadruplicate

Next, keeping potential biotechnological applications of the enzyme in mind, we characterized the hydrolytic activity of the α-fucosidase determining pH and temperature optima and temperature stability. The optimal pH was found to be 3.6, which is in line with the results published earlier [16] (Fig. 2a). The optimum temperature for a 30-min reaction was found to be 60 °C, but the enzyme was unstable at temperatures above 45 °C (Fig. 2b and c). It was concluded that considering protein stability as a relevant parameter the optimal temperature under the tested conditions was 45 °C.

Kinetic parameters were determined for pNP-hydrolysis with α-fucosidase; the assays were performed at the optimal conditions of pH 3.6 and 45 °C in quadruplicate using different pNP-fucose concentrations. The enzyme showed Michael-Menten kinetics. The value for the apparent K_m_ was determined as 0.39 mM, and V_max_ as 2.8 mmol/(mg*h). The K_m_ and V_max_ values have not been reported for *A. niger* α-fucosidase previously using pNP-fucose as substrate, but with methyl 2-*O*-α-l-fucopyranosyl-β-d-galactoside as substrate the K_m_ value has been determined as 0.083 mM and V_max_ as 16 μmol/(mg*h) [20]. Our results agree well with reports on the K_m_ values for other wild type α-fucosidases, ranging from 0.02 to 1.2 mM for pNP-fucose as substrate [21, 22].

### α-Fucosidase produces fucosyllactose in a transglycosylation reaction

Some family GH29 α-fucosidases are known to have also transglycosylation activity adding fucose units to a saccharide structure. In most earlier studies, pNP-fucose has been used as the substrate as the release of pNP can be measured spectrophotometrically [13, 17, 23, 24]. However, in this study we wanted to find out whether the enzyme can use unmodified sugars as substrates. The transglycosylation reaction was studied with various fucose and lactose concentrations, temperatures and reaction times. The enzyme concentration was varied between 0.15 and 15 U of α-fucosidase/ml. The reactions were analyzed for the production of fucosyllactose using LC-MS and a peak corresponding to the expected m/z of fucosyllactose was detected (data not shown). The analysis proved that the enzyme can indeed perform the transglycosylation reaction between fucose and lactose, although with a low yield. The optimal conditions for transglycosylation were 100 g/L fucose and 200 g/L lactose with 50 °C reaction temperature. Elongated incubation times between 7 and 11 days gave highest yields. In order to exclude the possibility of any enzyme independent side reactions, reactions with heat-inactivated α-fucosidase were prepared as controls.

The final transglycosylation was performed using optimal conditions with 0.15 U α-fucosidase/ml and a reaction time of 8 days. The reaction mixture was fractionated using gel chromatography and fractions were analyzed with LC-MS. Fractions with an m/z ratio of 487 corresponding to the mass of fucosyllactose and not containing other sugars as impurities were pooled and concentrated. This pooled sample had one major peak and minor amounts of alternative products with the same m/z ratio but eluting at slightly different times (see Fig. S2). It was suspected that these were products with different linkage types. The area of the major peak constituted approximately 80% of the area of all fucosyllactose peaks. When comparing the MS-data of the product with standards, this product was neither 2’-FL or 3-FL. The fractions with the main fucosyllactose product were pooled and concentrated. This product was purified further with LC for NMR analysis. The control reaction using heat-inactivated α-fucosidase yielded no product with the mass of fucosyllactose, so we concluded that the product was indeed produced by the α-fucosidase activity.

### Structure determination by NMR spectroscopy

**Fig. 3:**
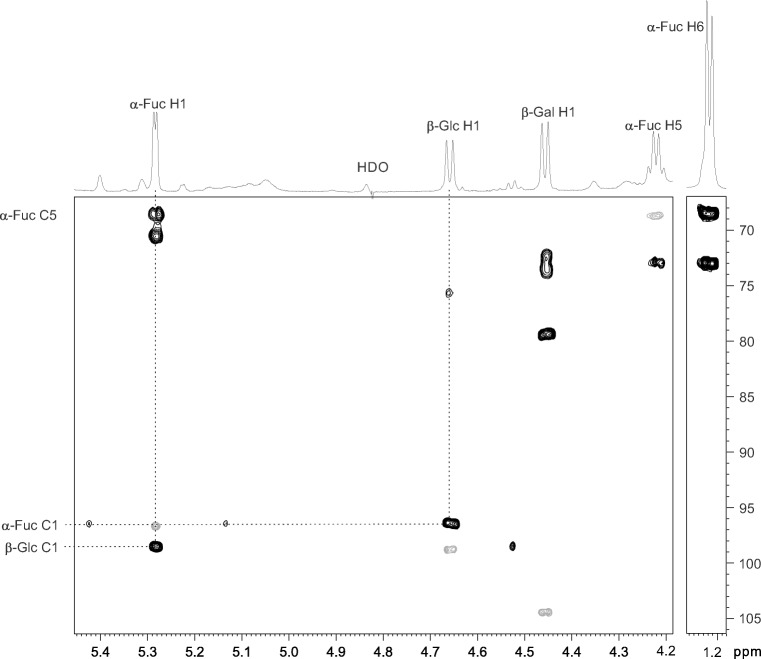
Expansions of the anomeric and methyl regions of the 1D ^1^H NMR and overlaid HMBC (black) and HSQC (gray) spectra of 1-fucosyllactose Galβ1-4Glcβ1-1αFuc. In the 1D spectrum, three anomeric signals of equal size are observed as well as the typical H5 and H6 signals of fucose. The dashed lines in the 2D spectrum indicate the HMBC connections over the Glcβ1-1αFuc glycosidic bond and the HSQC signals show the assignments of the anomeric carbon signals based on the corresponding protons

In order to identify the novel fucosyllactose, the structure was analyzed using NMR. The structural reporter group area of the ^1^H NMR spectrum of the trisaccharide (see 1D projection in Fig. 3) reveals three anomeric signals. These signals are of equal size, *i.e*. the normal approximately 30:70% division to two anomers was not observed, which suggested that the saccharide is not a reducing one. Two of the coupling constants of the anomeric signals are large (about 8 Hz), while one is small (3.6 Hz), suggesting that there are two β- and one α- anomer. In addition to the anomeric signals, a methyl signal was observed at about 1.05 ppm and a signal with a fine structure typical for fucose H5 was at about 4.05 ppm.

The ^1^H and ^13^C signals were assigned using standard 2D NMR techniques and the chemical shifts in Table S2 in Online Resource were determined from a pure shift HSQC spectrum (Fig. S3 in Online Resource). When the HSQC spectrum of the trisaccharide was compared to that of the acceptor lactose (see Fig. S3 in Online Resource) it was obvious that the galactose signals and most of the β-glucose signals were almost unaffected by the fucosylation, while the signals of α-glucose had disappeared. This together with the equal sizes of the three anomeric signals suggested that the structure of the trisaccharide is Galβ1-4Glcβ1-1αFuc. This was further confirmed by the HMBC signals between Glc H1 and Fuc C1 and Glc C1 and Fuc H1 (Fig. 3). Figure 3 also shows the HMBC signal from Fuc H6 to Fuc C5 and further to Fuc H1 confirming the assignments of the anomeric signals. In addition, a ROESY spectrum showed an NOE between Glc H1 and Fuc H1 (data not shown). Taken together, the NMR analysis of the trisaccharide revealed that the saccharide is 1-fucosyllactose or Galβ1-4Glcβ1-1αFuc.

### α-Fucosidase hydrolyzes its own transglycosylation product

According to our results, the transglycosylation reaction of α-fucosidase produced mainly 1-fucosyllactose, with only minor side products. We wanted to study, whether this α-fucosidase was capable of hydrolyzing its own transglycosylation product along with two common fucosyllactoses, 2’-FL and 3-FL. A hydrolysis reaction was performed using these substrates. As exact amounts of 1-FL were not known due to lack of standards, the amount was estimated based on peak areas in LC-MS and HPAEC-PAD. The portion of remaining sugars was determined by HPAEC-PAD using three technical replicates.

According to the results, 1-FL was clearly the best substrate, with 35% of FL hydrolyzed during the first hour and complete hydrolysis observed after 24 h. 2’-FL was also used as a substrate by the enzyme, but not as efficiently, reaching 16% and 82% hydrolysis within 1 h and 24 h, respectively. In contrast, 3-FL was a poor substrate with only 6% and 26% hydrolyzed within 1 h and 24 h, respectively. It is clear that 1-FL, which is also the product of transglycosylation reaction, is the preferred substrate of this enzyme from the substrates tested.

## Discussion

In this study, we characterized the recombinantly produced family GH29 α-fucosidase from *A. niger* CBS 513.88 in terms of its hydrolytic activity and its transglycosylation activity keeping potential industrial applications of the enzyme in mind. In particular, we were interested in testing its transglycosylation activity using lactose and free fucose as it would allow the production of fucosyllactose using excess lactose from dairy industries without the use of activated and potentially problematic fucose derivatives such as pNP-fucose or fucosyl-fluoride.

Besides the family GH29 α-fucosidase, *A. niger* CBS 513.88 contains another α-fucosidase activity belonging to family GH95 (by sequence similarity). So far, in all previous reports on *A. niger* α-fucosidases either an enzyme produced by a non-available and non-characterized *A. niger* isolate or a commercial enzyme preparation were used [17, 20, 23–25]. In fact, the enzyme preparation contained at least an α1,2- and α1,6-fucosidase activity [25]. The exact properties of the *A. niger* α-fucosidase remain partially elusive. Therefore, we have recombinantly produced the GH29 α-fucosidase.

For studying the hydrolytic activity, most studies have used either pNP-fucose [17, 23] or a sugar containing the Fuc-α1,2-Gal structure [20, 24] in the activity measurements. However, there are discrepancies between the studies on the enzyme’s ability to use pNP-fucose as substrate; some reports state that the enzyme is capable of utilizing pNP-fucose as substrate [17, 23, 24] while others clearly state that pNP-fucose is not hydrolyzed [20, 25]. Our data shows that the recombinant enzyme can hydrolyze pNP-fucose.

Furthermore, the enzyme preparations have been shown to hydrolyze the Fuc-α1,2-Gal linkage types and Fuc-α1,3-Glc while not being able to hydrolyze 3-FL or other Fuc-α1,3/4/6- linkages [17, 20]. These results led to the conclusion, that this is most likely a family GH29 enzyme. Our results show that the family GH29 α-fucosidase from *A. niger* CBS 531.88 has hydrolytic activity towards 2’-FL (Fuc-α1,2-linked to Gal) and slight activity on 3-FL (Fuc-α1,3-linked to Glc) which gives the impression that it might be the same enzyme as the one studied in most of the previous articles. In addition, the optimal pH and temperature and kinetic properties align well with the data from these studies [17, 20, 23, 24]. One study has been made where the purified fractions were screened with an activity assay using Fuc-asialo-agalacto-fetuin as substrate. This enzyme showed hydrolytic specificity towards Fuc-α1,6-GlcNAc and Fuc-α1,6-linkages in macromolecules and 1,2-α-L-fucosidase activity was eliminated in the purification procedure [25]. This seems to be a different enzyme from the rest of the studies, and due to its substrate specificity, it does not seem to belong to GH95 family either.

An activated form of fucose, pNP-fucose has been used as a substrate in the transglycosylation reactions with *A. niger* α-fucosidase. It is known that pNP acts as a good leaving group thus favoring the transglycosylation reaction. However, due to environmental concerns and costs, other substrates might be more attractive. Using recombinantly produced family GH29 α-fucosidase we were able to use free fucose and lactose as substrates in a transglycosylation reaction. To our knowledge, there are no previous reports using free fucose as substrate for fucosyllactose production with any enzyme. However, there are α-fucosidases, Mfuc5 from a soil metagenome and FgFCO1 from *Fusarium graminearum* that have been shown to use xyloglucan as fucose donor [8, 14, 26].

In previous reports using the supposed family GH29 enzyme, the transglycosylation product between pNP-fucose and glucose or GlcNac (or derivative thereof) has been a Fuc-α1,3-Glc containing structure [17, 23, 24]. However, our structural characterization of the transglycosylation product using NMR clearly show that in the transfucosylation of lactose using free fucose, the product is Galβ1-4Glcβ1-1αFuc or 1-FL where α-fucose is 1,1-linked to β-glucose, representing a novel fucosyllactose that so far has not been observed when using pNP-fucose as a donor. We might speculate that this unique structure is obtained due to the use of free fucose instead of an activated fucose donor. Furthermore, to our surprise, the enzyme could only use the β-anomer of lactose as a substrate as there was no signal of fucose linked to α-glucose being present.

As we managed to produce a novel fucosyllactose, we wanted to also study whether the enzyme could use this 1-FL as a substrate, or whether it was accumulating into the production media due to the lack of the hydrolytic reaction. Surprisingly we found out, that among the fucosyllactoses studied, 1-FL was the preferred substrate of the enzyme. As this sugar has not been produced previously, there is no data available, whether it is also used as a substrate by other α-fucosidases. Non-reducing oligosaccharides often exhibit beneficial properties, and this is, to our knowledge, the first report of a non-reducing fucose-containing oligosaccharide. This novel oligosaccharide could also be used in an array of oligosaccharide binding studies. The more oligosaccharide structures there are available, the better arrays could be produced. Furthermore, this new product could also be used as a standard when studying unknown enzymatic activities. In this context it is important to note, that a fucosyllactose other than the well-known 2’-FL and 3-FL was found as a translycosylation product in another study [14].

As a summary, the GH29 α-fucosidase from *A. niger* CBS 531.88 has an optimal pH of 3.6 and optimal temperature of 45 °C for incubation times of several hours. Under these conditions, the enzyme is able to perform a transglycosylation reaction between fucose and lactose, forming a novel, non-reducing fucosyllactose structure, namely Galβ1-4Glcβ1-1αFuc or in short 1-FL. This enzyme also prefers 1-FL as substrate in hydrolysis over 2’-FL and 3-FL.

## Electronic supplementary material

ESM 1 (PDF 439 kb)
