# Supplementary material for: Production and characterization of Aspergillus niger GH29 family α-fucosidase and production of a novel non-reducing 1-fucosyllactose
Source: Glycoconj J. 2019 Dec 2;37(2):221–9. doi: 10.1007/s10719-019-09896-w (PMC7083800; doi:10.1007/s10719-019-09896-w)
Supplement: Supplementary file 1 — (PDF 439 kb) [file 10719_2019_9896_MOESM1_ESM.pdf]

Production and characterization of *Aspergillus niger* GH29 family α-fucosidase and production of a novel non-reducing 1-fucosyllactose

Glycoconjugate journal

Usvalampi, A., Ruvalcaba Medrano, M., Maaheimo, H., Salminen, H., Tossavainen, O., Frey, A.D.
Corresponding author: Anne Usvalampi
Department of Bioproducts and Biosystems, Aalto University School of Chemical Engineering, P.O.Box 16100, Espoo, Finland, anne.usvalampi@aalto.fi

| | | | | | | | | | |
|---|---|---|---|---|---|---|---|---|---|
| 10 | 20 | 30 | 40 | 50 | 60 | 70 | 80 | 90 | 100 |
| VPLGLKLIIQ | SEFVSLDSYF | NNKAFGVYPG | QTSFDALNQS | YPDPSIIGIN | GTYTSTHTGV | VYDFPGYRGP | DLPDNVVCEG | QTINVPRDQY | FSASMLVTSD |
| 110 | 120 | 130 | 140 | 150 | 160 | 170 | 180 | 190 | 200 |
| VQLSTVSGNV | TYTYSDDSTL | VSELRSLPWW | AFLTINRGEI | IFPYRYTHND | TNFNTSHIFE | YSAALDPNKT | LHSITLPVTT | NTTTGRLHIF | SVSLWKSTAP |
| 210 | 220 | 230 | 240 | 250 | 260 | 270 | 280 | 290 | 300 |
| SAQVQFVRPT | QKWTESGNQI | VEVTINNPGS | SCIAGPGVNL | SLSMPGIRTL | EPANVKRLCP | GDQKRVNIGI | NGTANGTATV | ILNYTHSIQH | QTYPHPLSLG |
| 310 | 320 | 330 | 340 | 350 | 360 | 370 | 380 | 390 | 400 |
| LTEWTSDLSS | LSQHESPEWF | DDAKFGIMIH | WGPYSVPGWG | NSTPYESYAE | WFWWYTTHRA | ADKSDTYDYR | LRTFGPTWNY | DDSFPSFTAA | NFSPKAWVDL |
| 410 | 420 | 430 | 440 | 450 | 460 | 470 | 480 | 490 | 500 |
| ISASGAKYFV | LTTKHHDGFA | LFDTQNTTNR | SSLHYGPHRD | LVSELFTASS | IHHPTLKRGT | YFSLPEWFNP | DFGPYGFAEL | PGNTSTSWPG | IIARNPYTGL |
| 510 | 520 | 530 | 540 | 550 | 560 | 570 | 580 | 590 | 600 |
| NEPYTGRIPV | TDFITDVMVP | QMTILAHNYS | TDIMWCDCGA | ANGTASFASE | WFNSARADNR | QVAINSRCGV | AEVSDFDTPE | YATFSSAQLR | KWESNMGMDP |
| 610 | 620 | 630 | 640 | 650 | 660 | 670 | 680 | 690 | 700 |
| YSYGFNRATA | ERSYMNASTV | VRDLVDMVSK | NGNFLLDVGP | RADGSIVERE | ERELRRAGEW | IRGHGEAVFG | TRVWWVRSEA | VGVTGDGGDV | GVRFTQTNEA |
| 710 | 720 | 730 | 740 | 750 | 760 | 770 | 780 | 790 | |
| FYLLFLGDPG | KKVFVDASVP | LLRGDRVVVV | GGDGEREVEV | EWEGSATEGF | TFRVPEGVWD | GEEFCWVLKI | VYLAHHHHHH | | |

***Fig S1.*** *MALDI-TOF MS/MS result of Endo H treated α-fucosidase. The results were analyzed with ProteinScape software (Bruker). The sequence coverage was 61 % and number of peptides found was 51. Fixed modifications: HexNAc linked to amino acids 391, 426, 429, 483 and 616. Variable modifications: Carbamidomethyl and Propionamide (C), HexNAc (N).*

***Table S1.*** *MALDI-TOF MS/MS result of Endo H treated α-fucosidase.*

| m/z meas. | Δ m/z [ppm] | Δ m/z [Da] | Score[1] | P[2] | Sequence | Modification | Range |
|---|---|---|---|---|---|---|---|
| 2094.0119 | -7.89 | -0.0165 | 26.6646 | 0 | R.GPDLPDNVVCEGQTINVPR.D | Propionamide: 10 | 69-87 |
| 1503.8041 | -4.39 | -0.0066 | 51.3314 | 0 | R.SLPWWAFLTINR.G | | 126-137 |
| 994.533 | -2.66 | -0.0026 | 61.3394 | 0 | R.GEIIFPYR.Y | | 138-145 |
| 1229.6929 | -9.11 | -0.0112 | 71.8164 | 0 | R.LHIFSVSLWK.S | | 187-196 |
| 1290.6786 | -1.14 | -0.0015 | 14.8912 | 0 | K.STAPSAQVQFVR.P | | 197-208 |
| 1744.923 | -6.34 | -0.0111 | 41.7465 | 1 | K.STAPSAQVQFVRPTQK.W | | 197-212 |
| 3768.7559 | -29.84 | -0.1125 | 30.0576 | 0 | K.WTESGNQIVEVTINNPGSSCIAGPGVNLSLSMPGIR.T | Propionamide: 20 | 213-248 |
| 1027.6088 | 18.81 | 0.0193 | 15.7759 | 1 | R.TLEPANVKR.L | | 249-257 |
| 973.5667 | 80.46 | 0.0783 | 21.7862 | 1 | R.LCPGDQKR.V | Carbamidomethyl: 2 | 258-265 |
| 1304.5641 | -8.61 | -0.0112 | 32.0305 | 1 | R.AADKSDTYDYR.L | | 360-370 |
| 919.3928 | 14.79 | 0.0136 | 17.4488 | 0 | K.SDTYDYR.L | | 364-370 |
| 2799.8435 | -42.07 | -0.3978 | 50.1029 | 0 | R.TFGPTWNYDDSFPSFTAANFSPK.A | HexNAc: 19 | 373-395 |
| 2597.1185 | -16.73 | -0.0434 | 33.9720 | 0 | R.TFGPTWNYDDSFPSFTAANFSPK.A | | 373-95 |
| 1217.6763 | 19.58 | 0.0238 | 71.4306 | 0 | K.AWVDLISASGAK.Y | | 396-407 |
| 871.5061 | 15.74 | 0.0137 | 56.5386 | 0 | K.YFVLTTK.H | | 408-414 |
| 2077.0718 | 64.92 | 0.1348 | 37.4770 | 0 | K.HHDGFALFDTQNTTNR.S | HexNAc: 12 | 415-430 |
| 2280.0544 | 16.69 | 0.0381 | 59.0289 | 0 | K.HHDGFALFDTQNTTNR.S | HexNAc: 12, 15 | 415-430 |
| 1053.5426 | 19.12 | 0.0201 | 13.8693 | 0 | R.SSLHYGPHR.D | | 431-439 |
| 3029.5079 | -16.93 | -0.0513 | 41.8732 | 1 | R.SSLHYGPHRDLVSELFTASSIHHPTLK.R | | 431-457 |

| | | | | | | | |
|---|---|---|---|---|---|---|---|
| 1995.0911 | 18.31 | 0.0365 | 53.1468 | 0 | R.DLVSELFTASSIHHPTLK.R | | 440-457 |
| 2151.1987 | 20.00 | 0.0430 | 26.7589 | 1 | R.DLVSELFTASSIHHPTLKR.G | | 440-458 |
| 4350.8343 | -55.80 | -0.2428 | 30.2034 | 1 | K.RGTYFSLPEWFNPDFGPYGFAELPGNTSTSWPGIIAR.N | HexNAc: 26 | 458-494 |
| 4194.7763 | -47.60 | -0.1997 | 26.9779 | 0 | R.GTYFSLPEWFNPDFGPYGFAELPGNTSTSWPGIIAR.N | HexNAc: 25 | 459-494 |
| 1481.6936 | -5.62 | -0.0083 | 57.4094 | 0 | R.NPYTGLNEPYTGR.I | | 495-507 |
| 787.4558 | 17.43 | 0.0137 | 34.7147 | 0 | R.QVAINSR.C | | 561-567 |
| 2550.1741 | 11.29 | 0.0288 | 45.7482 | 0 | R.CGVAEVSDFDTPEYATFSSAQLR.K | Carbamidomethyl: 1 | 568-590 |
| 2564.1291 | -12.43 | -0.0319 | 66.2118 | 0 | R.CGVAEVSDFDTPEYATFSSAQLR.K | Propionamide: 1 | 568-590 |
| 2691.9964 | -96.40 | -0.2595 | 44.1666 | 1 | R.CGVAEVSDFDTPEYATFSSAQLRK.W | Propionamide: 1 | 568-591 |
| 2081.8647 | -9.47 | -0.0197 | 53.4217 | 1 | R.KWESNMGMDPYSYGFNR.A | | 591-607 |
| 1953.7719 | -8.98 | -0.0175 | 42.4770 | 0 | K.WESNMGMDPYSYGFNR.A | | 592-607 |
| 1330.6854 | 41.08 | 0.0547 | 18.9146 | 0 | R.SYMNASTVVR.D | HexNAc: 4 | 613-622 |
| 2218.1159 | 19.34 | 0.0429 | 10.9567 | 1 | R.SYMNASTVVRDLVDMVSK.N | HexNAc: 4 | 613-630 |
| 906.485 | 27.48 | 0.0249 | 28.6895 | 0 | R.DLVDMVSK.N | | 623-630 |
| 2089.056 | -8.93 | -0.0187 | 22.8787 | 1 | R.DLVDMVSKNGNFLLDVGPR.A | | 623-641 |
| 1201.6255 | -5.74 | -0.0069 | 71.7611 | 0 | K.NGNFLLDVGPR.A | | 631-641 |
| 846.4582 | 31.46 | 0.0266 | 23.5537 | 0 | R.ADGSIVER.E | | 642-649 |
| 1260.6103 | -6.00 | -0.0076 | 34.2644 | 1 | R.ADGSIVEREER.E | | 642-652 |
| 887.4833 | -1.48 | -0.0013 | 36.6031 | 1 | R.RAGEWIR.G | | 656-662 |
| 731.3767 | -9.30 | -0.0068 | 46.7806 | 0 | R.AGEWIR.G | | 657-662 |
| 1030.5032 | -3.17 | -0.0033 | 60.6507 | 0 | R.GHGEAVFGTR.V | | 663-672 |

[1] ProteinScape Theoretical Digest score.
[2] Partials (missed cleavages).

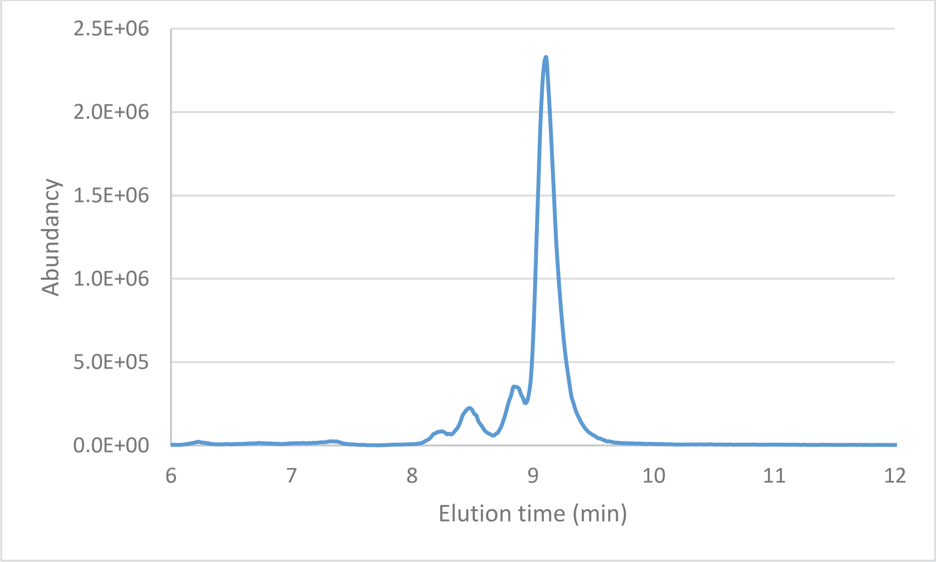


***Fig S2.*** *Abundancy of the product from transglycosylation reaction corresponding to the structure* $C_{18}H_{32}O_{15}$*, i.e. fucosyllactose. The transglycosylation reaction was fractionated with gel chromatography and the trisaccharide-containing fractions were pooled and analyzed on LC-MS. Besides the major peak at 9.1 minutes there were some minor peaks that most likely correspond to fucosyllactoses of different linkage types. The following settings were used: Source gas temperature 300 °C, drying gas 10 l/min, nebulizer 20 psig, capillary voltage 3000 V.*

***Table S2.*** *$^{1}H$ and $^{13}C$ chemical shifts of 1-fucosyllactose Galβ1-4Glcβ1-1αFuc in $D_2O$ at 22 ºC.*

| | $^{1}H$ chemical shift (ppm) | $^{13}C$ chemical chift (ppm) |
|---|---|---|
| Gal 1 | 4.456 | 104.2 |
| 2 | 3.545 | 72.3 |
| 3 | 3.666 | 73.8 |
| 4 | 3.926 | 69.8 |
| 5 | 3.727 | 76.7 |
| 6 | 3.773 | 62.3 |
| | | |
| Glc 1 | 4.658 | 98.5 |
| 2 | 3.423 | 73.7 |
| 3 | 3.674 | 75.5 |
| 4 | 3.701 | 79.5 |
| 5 | 3.598 | 76.2 |
| 6 | 3.971/3.826 | 61.2 |
| | | |
| Fuc 1 | 5.283 | 96.5 |
| 2 | 3.838 | 68.6 |
| 3 | 3.913 | 70.7 |
| 4 | 3.833 | 73.0 |
| 5 | 4.220 | 68.5 |
| 6 | 1.216 | 16.6 |

The $^{1}H$ and $^{13}C$ chemical shifts were referenced to internal acetone, 2.225 and 31.55 ppm, respectively.

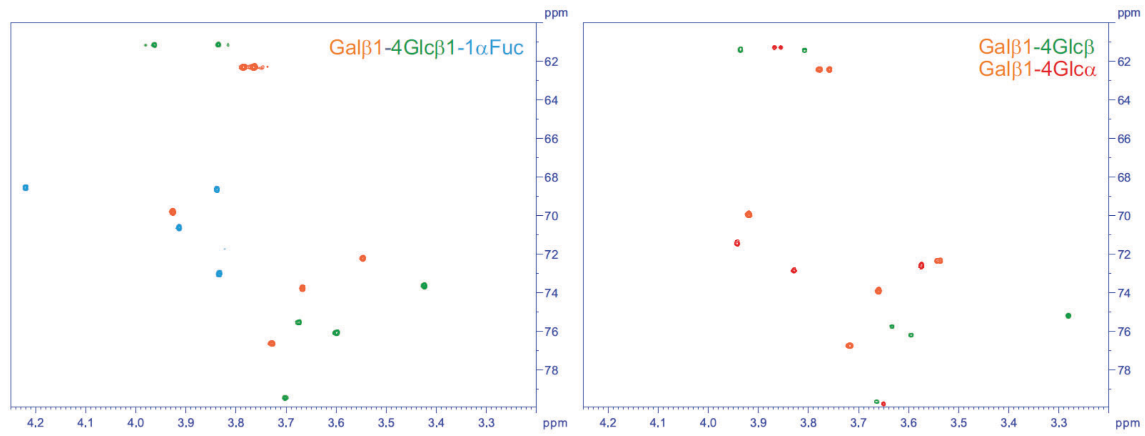


***Fig S3.*** *Ring proton/carbon area of pure shift HSQC spectra of 1-fucosyllactose (left) and lactose (right). When the spectrum of 1-fucosyllactose is compared to that of lactose, it is obvious that the signals of β-galactose (orange) and β-glucose (green) are virtually unchanged, while the signals of α-glucose (red) have disappeared. Fucose signals are shown in light blue.*
